# Supplementary material for: Hydroxychloroquine and a low antiresorptive activity bisphosphonate conjugate prevent and reverse ovariectomy-induced bone loss in mice through dual antiresorptive and anabolic effects
Source: Bone Res. 2024 Sep 5;12:52. doi: 10.1038/s41413-024-00352-6 (PMC11375055; doi:10.1038/s41413-024-00352-6)
Supplement: Supplementary file 1 — Hydroxychloroquine and a low antiresorptive activity bisphosphonate conjugate prevent and reverse ovariectomy-induced bone loss in mice through dual antiresorptive and anabolic effects [file 41413_2024_352_MOESM1_ESM.doc]

**Supporting Information**

Hydroxychloroquine and a low antiresorptive activity bisphosphonate conjugate prevent and reverse ovariectomy-induced bone loss in mice through dual antiresorptive and anabolic effects

Zhenqiang Yao^1^,*, Akram Ayoub^1^, Venkatesan Srinivasan^2^, Jun Wu^1^, Churou Tang^1,3^, Rong Duan^1^, Aleksa Milosavljevic^2^, Lianping Xing^1^, Frank H Ebetino^2,4^, Alison Frontier^2^, Brendan F. Boyce^1^,*.

**Affiliations:**

^1^ Department of Pathology and Laboratory Medicine, University of Rochester Medical Center, Rochester, NY 14642, USA; ^2^ Department of Chemistry, University of Rochester, Rochester NY14627, USA; ^3^ School of Arts and Science, University of Rochester, Rochester NY14627, USA; ^4^ BioVinc, LLC, 2265 E Foothill Blvd, Pasadena, CA 91107, USA.

Corresponding authors: Zhenqiang Yao, PhD, Telephone: 585-275-8532, E-mail: Zhenqiang_Yao@urmc.rochester.edu; and Brendan F. Boyce M.D, Telephone: (585) 275-5837, Fax: (585) 276-2047, E-mail: Brendan_Boyce@urmc.rochester.edu, Department of Pathology and Laboratory Medicine, 601 Elmwood Ave, Box 626, Rochester, NY 14642, USA.

| **Table of Contents** | **Page** |
| --- | --- |
| 1. Chemical Synthesis of **HABP-HCQ** | S2 |
| 2. **HABP-HCQ** NMR Studies | S5 |
| 3. **HABP-HCQ** High-Resolution Mass Spectrometry (HRMS) Studies | S7 |
| 4. **HABP-HCQ** inhibits osteoclast formation in mice with lost bone caused by OVX | S9 |
| 5. NMR Spectra | S10-19 |
|  |  |
|  |  |

**General Remarks**

All vacuum/argon flushes and flame drying techniques were performed using a Schlenk line, along with septa and needles (no Schlenk flasks or multi-neck flasks were used, unless specifically stated). Reagents were used as obtained from commercial suppliers without further purification, unless otherwise noted. Tetrahydrofuran (THF), diethyl ether, dichloromethane, and toluene were dried using a solvent purification system, and kept under argon before use. Methanol and dimethylformamide (DMF) were purchased from Fisher and dried by the addition of vacuum/flame-dried 4 Å molecular sieves (typically with a ~ 2 cm layer of ball-shaped sieves per solvent bottle, over at least three days). The solvents were used under air. 4 Å and 5 Å molecular sieves were purchased from Aldrich and dried by heating with a standard 700 W kitchen microwave (4-6x 1.5-2 min cycles, with manual shaking in between cycles, open to air), followed by cooling to room temperature under vacuum (< 1 Torr). Screw-top tubes and flasks were made of heavy glass walls, to withstand higher pressures than the common round bottom flasks (see CG-1880 on Chemglass). Celite 545 was purchased from EMD. ACS-grade hexanes, toluene, ethyl acetate, and DCM were used for column chromatography. Thin-layer chromatography (TLC) was performed on pre-coated silica gel 60 F254 glass-supported plates from EMD, and visualization was performed with a UV lamp followed by staining with an indicated solution. Column chromatography was carried out on EM Science silica gel (60 Å pore size, 230-400 mesh). Deuterated solvents were purchased from Cambridge Isotope Laboratories. Potassium carbonate was flame-dried in a flask under vacuum, let to cool to room temperature, filled with argon, and as such added to bottles of deuterated chloroform to remove any acids formed due to the spontaneous decomposition of chloroform in the presence of light and oxygen. For the preparation of NMR samples, about 0.70-0.75 mL of CDCl_3_/K_2_CO_3_, measured with a syringe and needle, was filtered through a small pad of cotton (placed in a Pasteur pipette), and a pipette bulb was used to push out the residual chloroform from the cotton pad. Other deuterated solvents were used as received.

^1^H, ^13^C, and ^31^P NMR spectra were recorded at room temperature (unless otherwise stated) on a 500 MHz Bruker Avance spectrometer or a 400 MHz Bruker Avance spectrometer, using TopSpin v1.3, and processed in MNova v14.2.1. Chemical shifts are given in parts per million (ppm) referenced to solvent residual resonance. NMR data are reported as chemical shift, multiplicity (s = singlet, d = doublet, t = triplet, q = quartet, p = pentet/quintet, m = multiplet, and any combination of d, t, q, and p e.g., dt = doublet of triplets), coupling constants (*J*) given in Hz, and integration.

High-resolution mass spectra (HRMS) were measured at the University of Rochester Mass Spectrometry Resource Lab by Kevin Welle.

1. **Chemical Synthesis of HABP-HCQ**

Synthesis of **TBSHCQ**

An oven-dried round bottom flask was charged with a suspension of hydroxychloroquine sulfate in anhydrous dichloromethane (8 g, 18.43 mmol in 50 mL). Neat triethylamine (7.7 mL, 55.29 mmol) was added, followed by portion-wise addition of *tert*-butylchlorodimethylsilane (3.34 g, 22.116 mmol, added in six portions over one hour). The white suspension was stirred at room temperature for 2 d. The reaction mixture was diluted with dichloromethane (200 mL) and washed with an aqueous solution of sodium bicarbonate (3 x 30 mL). The organic layer was washed with brine (1 x 50 mL), dried over anhydrous sodium sulfate, filtered, and concentrated under reduced pressure. The crude mixture was purified by column chromatography on silica (1% Et_3_N in DCM) to yield 6.2 g (75 %) of **TBSHCQ** as a white powder.

R*_f_* (SiO_2_) 0.3 (1% Et_3_N/DCM, UV)

^1^H-NMR (400 MHz, CDCl_3_). δ 8.44 (d, *J* = 4 Hz, 1H), 7.94-7.82 (m, 2H), 7.33 (dd, *J_1_* = 8 Hz, J_2_ = 4 Hz, 1H), 6.36 (*J* = 4 Hz, 1H), 3.84-3.69 (m, 3H), 2.81-2.61 (m, 6H), 1.89-1.75 (m, 1H), 1.74-1.59 (m, 3H), 1.30 (d, *J* = 8 Hz, 3H), 1.08 (t, *J* = 8 Hz, 3H), 0.83 (s, 9H), 0.01 (s, 6H).

^13^C NMR (126 MHz, CDCl_3_) δ 152.16, 149.53, 149.10, 134.86, 128.97, 125.11, 121.28, 117.43, 99.39, 61.85, 55.53, 53.95, 48.45, 34.51, 26.07, 24.24, 20.37, 18.45, 11.71, -5.18.

Thermo-MS (ESI) m/z (M + H^+^) Cald for C_24_H_40_ClN_3_OSi: 451.13 and 452.13, found: 451.1 and 452.1.

Synthesis of tetraethyl **HABP** chloroformate

An oven-dried round bottom flask was charged with a solution of tetraethyl (aminomethylene)bis(phosphonate) in anhydrous tetrahydrofuran (12 g, 39.5 mmol in 60 mL). Succinic anhydride (4.8 g, 47.9 mmol) was added, and the contents were refluxed at 70 °C for 3 h. The reaction mixture was cooled to 0 °C, then borane dimethyl sulfide (7.5 mL, 79 mmol) was added dropwise (effervescence was observed during the addition), and the contents were gradually warmed to room temperature (flash was kept in the ice bath that was allowed to melt) and stirred for 2 h. After cooling to 0 °C, the contents were carefully quenched with methanol (effervescence observed) and stirred at room temperature for 30 min. The reaction mixture was concentrated under reduced pressure and the crude mixture was purified by column chromatography on silica (0 to 10 % methanol in ethyl acetate) to yield 10.76 g (70 %) of tetraethyl **HABP** as a colorless oil which slowly solidified to colorless solid upon standing.

R*_f_* (SiO_2_) 0.5 (10 % methanol in dichloromethane, KMnO_4_).

^1^H-NMR (400 MHz, CD_3_OD) δ 5.09 (t, *J* = 22.4 Hz, 1H), 4.23 – 4.05 (m, 8H), 3.57 (t, *J* = 5.6 Hz, 2H), 2.38 (t, *J* = 10 Hz, 2H), 1.91 – 1.72 (m, 2H), 1.39 – 1.25 (m, 12H).

^13^C-NMR (125 MHz, CD_3_OD) δ 174.56, 64.35 (d, *J* = 16.25 Hz), 61.44, 44.00 (t, *J* = 250 Hz), 32.30, 29.02, 16.03 (d, *J* =10 Hz).

^31^P-NMR (162 MHz, CD_3_OD) δ 14.93.

Thermo-MS (ESI) m/z (M + Na^+^) Calcd for C_13_H_29_NO_8_P_2_Na: 412.13 Found: 412.4.

A flame dried round bottom flask was charged with a suspension of anhydrous potassium carbonate (previously kept in an oven at 180 °C for at least 24 h) in anhydrous dichloromethane (3.85 g in 30 mL). After cooling to 0 °C, triphosgene (2.76 g, 9.31 mmol) was added, followed by *N,N*-diisopropylethylamine (DIPEA, 16 μL, 0.093 mmol). After stirring for 30 mins, a solution of tetraethyl **HABP** in anhydrous dichloromethane (3.62 g in 5 mL) was added over a period of 10 min. The reaction mixture was stirred at 0 °C for 3 h. The reaction mixture was cannula-filtered into a pre-weighed round bottom flask, and the mixture was concentrated under reduced pressure (in a fume hood) to yield 4.2 g of tetraethyl **HABP** chloroformate as a pale-yellow oil. The compound was used immediately in the next step.

^1^H-NMR (400 MHz, CDCl_3_) δ 6.36 (d, *J* = 9.6 Hz, 1H), 5.34 – 4.87 (m, 1H), 4.36 (t, *J* = 6.4 Hz, 2H), 4.31 – 4.08 (m, 8H), 2.39 (t, *J* = 7.2 Hz, 2H), 2.15 – 2.02 (m, 2H), 1.39 – 1.25 (m, 12H).

^31^P-NMR (162 MHz, CDCl_3_) δ 14.53.

Synthesis of **HABP-HCQ**

A flame-dried round bottom flask was charged with a suspension of freshly activated potassium carbonate in anhydrous dichloromethane (3.85 g in 30 mL), followed by the addition of **TBSHCQ** (4.3 g 9.31 mmol). After cooling to 0 °C, a solution of tetraethyl **HABP** chloroformate in anhydrous dichloromethane (4.2 g, 9.31 mmol in 6 mL) was added dropwise over a period of 15 mins. After stirring at 0 °C for 1.5 h, the solution was cannulated into another flame-dried flask maintained at 0 °C. Neat bromotrimethylsilane (9.8 mL, 74.48 mmol) was added dropwise and the contents were gradually warmed to room temperature (without removing the ice bath) and stirred for 2.5 d. After concentrating the reaction mixture under reduced pressure (in a fume hood), the crude mixture was redissolved in 10 mL methanol and concentrated. This process was repeated twice to yield a pale-yellow foam. The foam was washed with anhydrous acetonitrile (8 x 25 mL sonicated for 3 mins for each wash) and dried under reduced pressure at 35 °C for a day to yield 5.2 g (91 %) of **HABP-HCQ** as a pale-yellow powder. The structure of **HABP-HCQ** was determined in H_2_O/D_2_O to be the salt shown above. NMR spectrum of a **HABP-HCQ** sample mixed with **HCQ sulfate** only shows one set of peaks belonging to **HCQ** (both ^1^H and ^13^C NMR). ^1^H NMR spectrum of **HABP-HCQ** shows 1:1 ratio of the dication **HCQ** to the dianion **HABP**.

^1^H-NMR (400 MHz, D_2_O) δ 8.13 (d, *J* = 8 Hz, 1H), 8.06 (d, *J* = 8 Hz, 1H), 7.65 (s, 1H), 7.46 (d, *J* = 8 Hz, 1H), 6.71 (d, *J* = 8 Hz, 1H), 4.50 (t, *J* = 22.4 Hz, 1H), 4.08 – 3.92 (m, 1H), 3.72 (t, *J* = 4.6 Hz, 2H), 3.63 (2H, *J* = 7.2 Hz, 2H), 3.19 – 3.01 (m, 6H), 2.31 (t, *J* = 8 Hz, 2H), 2.02 – 1.85 (m, 2H), 1.74 – 1.56 (m, 4H), 1.26 (d, *J* = 7.2 Hz, 3H), 1.11 (t, *J* = 7.6 Hz, 3H).

^13^C-NMR (125 MHz, D_2_O) δ 179.03, 155.97, 142.88, 139.91, 138.65, 127.93, 124.83, 119.58, 115.75, 99.37, 56.02, 54.53, 52.79, 50.70 (t, *J* = 135 Hz), 50.26, 49.11, 47.84, 32.59, 30.82, 20.74, 19.51, 18.32, 8.70 (d, *J* = 50 Hz).

^31^P-NMR (162 MHz, D_2_O) δ 12.22, 10.66.

1. **HABP-HCQ NMR Studies**

*

*

**HABP-HCQ + HCQ sulfate (D_2_O)**

**HABP-HCQ (D_2_O)**

**HCQ sulfate (D_2_O)**

*

*

**Fig. S1.** Stacked ^1^H NMR spectra of **HCQ sulfate** (red), **HABP-HCQ** (green), and a sample prepared by mixing **HCQ sulfate** with **HABP-HCQ**, in D_2_O. Peaks labeled with * belong to **HABP**.

**HCQ sulfate (D_2_O)**

**HABP-HCQ**

**(D_2_O)**

**HABP-HCQ + HCQ sulfate(D_2_O)**

**Fig. S2.** Stacked ^13^C NMR spectra of **HCQ sulfate** (red), **HABP-HCQ** (green), and a sample prepared by mixing **HCQ sulfate** with **HABP-HCQ**, in D_2_O.

1. **HABP-HCQ High-Resolution Mass Spectrometry (HRMS) Studies**

**Fig. S3.** HRMS of **HCQ** **sulfate**.

**Fig. S4.** HRMS of **HABP** and a tentative assignment for the most abundant peak.

**Fig. S5.** HRMS of **HABP-HCQ**.

HRMS of **HABP-HCQ** is in agreement with the proposed ionic structure. No peaks of any covalent adducts of **HABP** and **HCQ** were detected.

1. HABP-HCQ inhibits osteoclast formation in mice with bone loss caused by OVX.


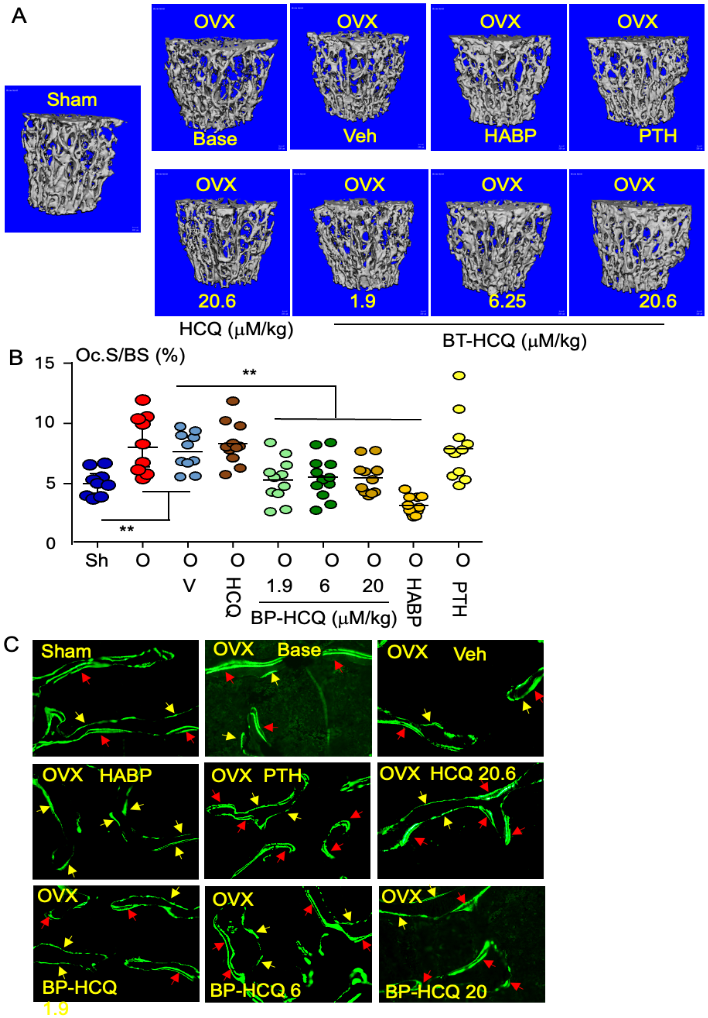


**Fig. S6.** Low-dose HABP-HCQ restores the lost bone caused by OVX by inhibiting bone resorption and maintaining high levels of bone formation. (A) Micro-CT-scanned L1 vertebral images, (B) Osteoclast surfaces on trabecular bone surface (Oc.S/BS) were quantified on TRAP-stained L4 vertebrae from mice, and (C) Calcein-labelled imaging in undecalcified L1 vertebral bone (red arrows = calcein double labelled surface, yellow arrows = calcein single labelled surface), in mice, as in Fig. 5. *p<0.05, **p<0.01, one-way ANOVA+/Dunnett test.

1. **NMR Spectra**

**
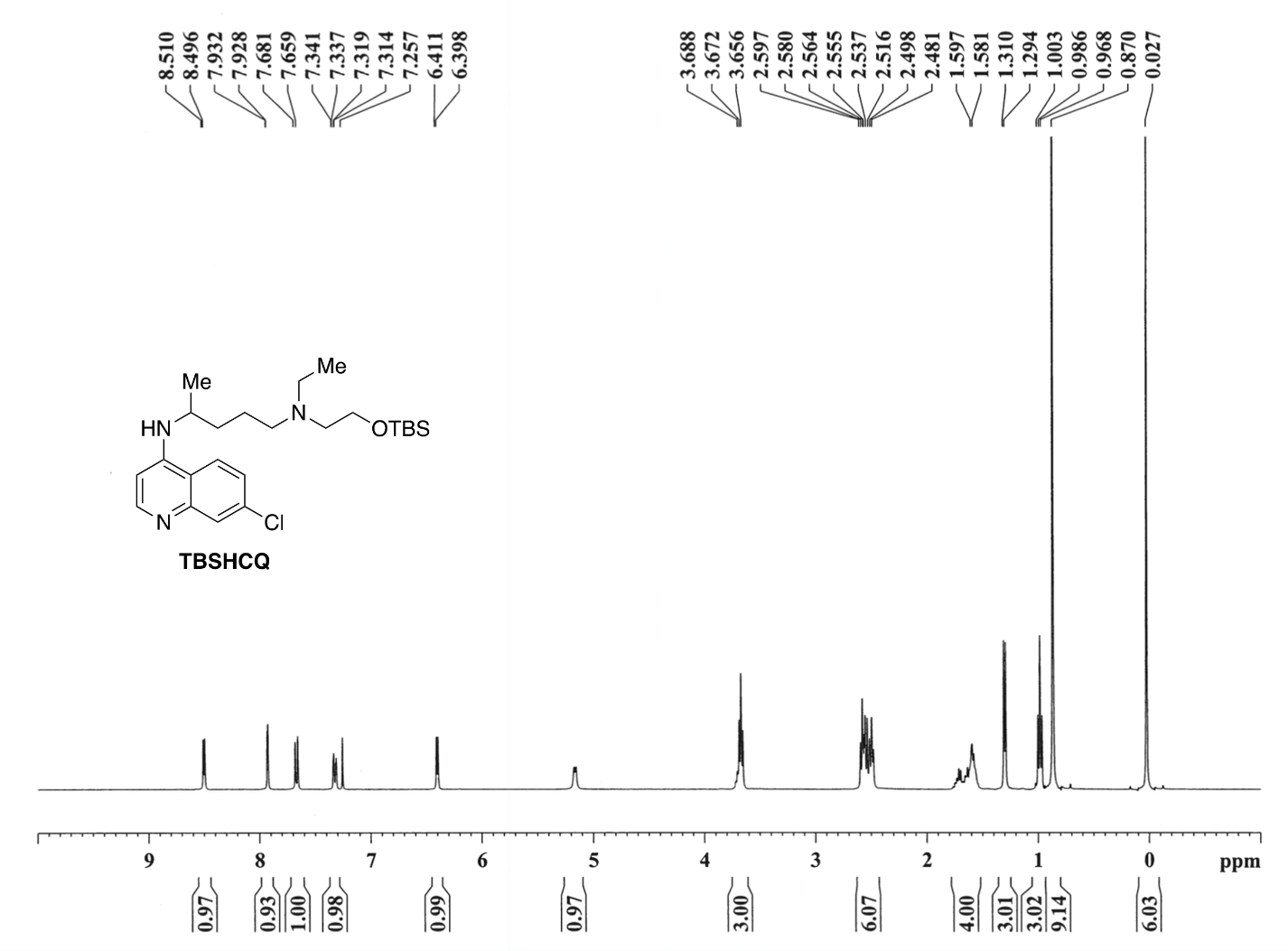
**

^1^H NMR Spectrum (CDCl_3_)

^13^C NMR Spectrum (CDCl_3_)


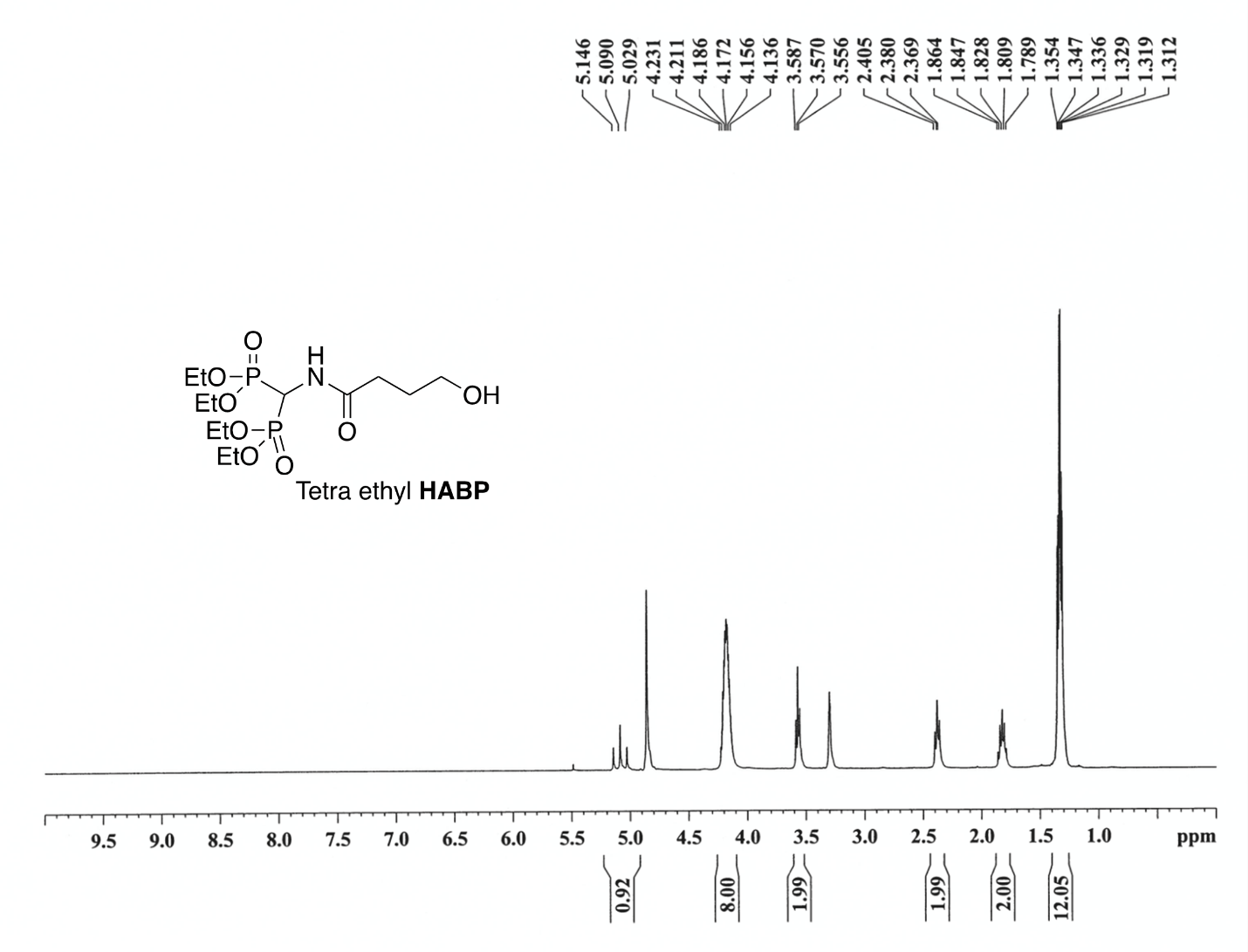


^1^H NMR Spectrum (CD_3_OD)


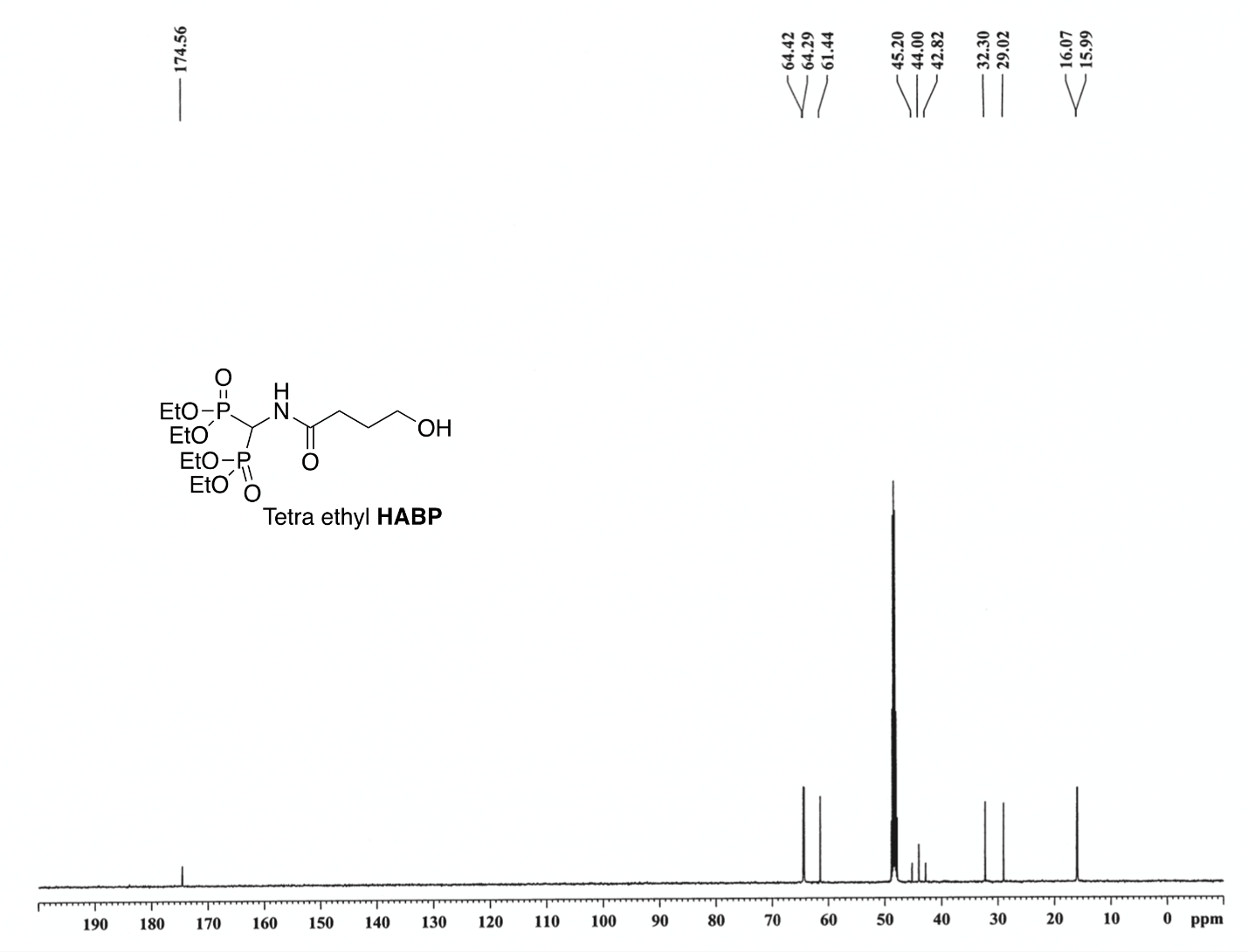


^13^C NMR Spectrum (CD_3_OD)


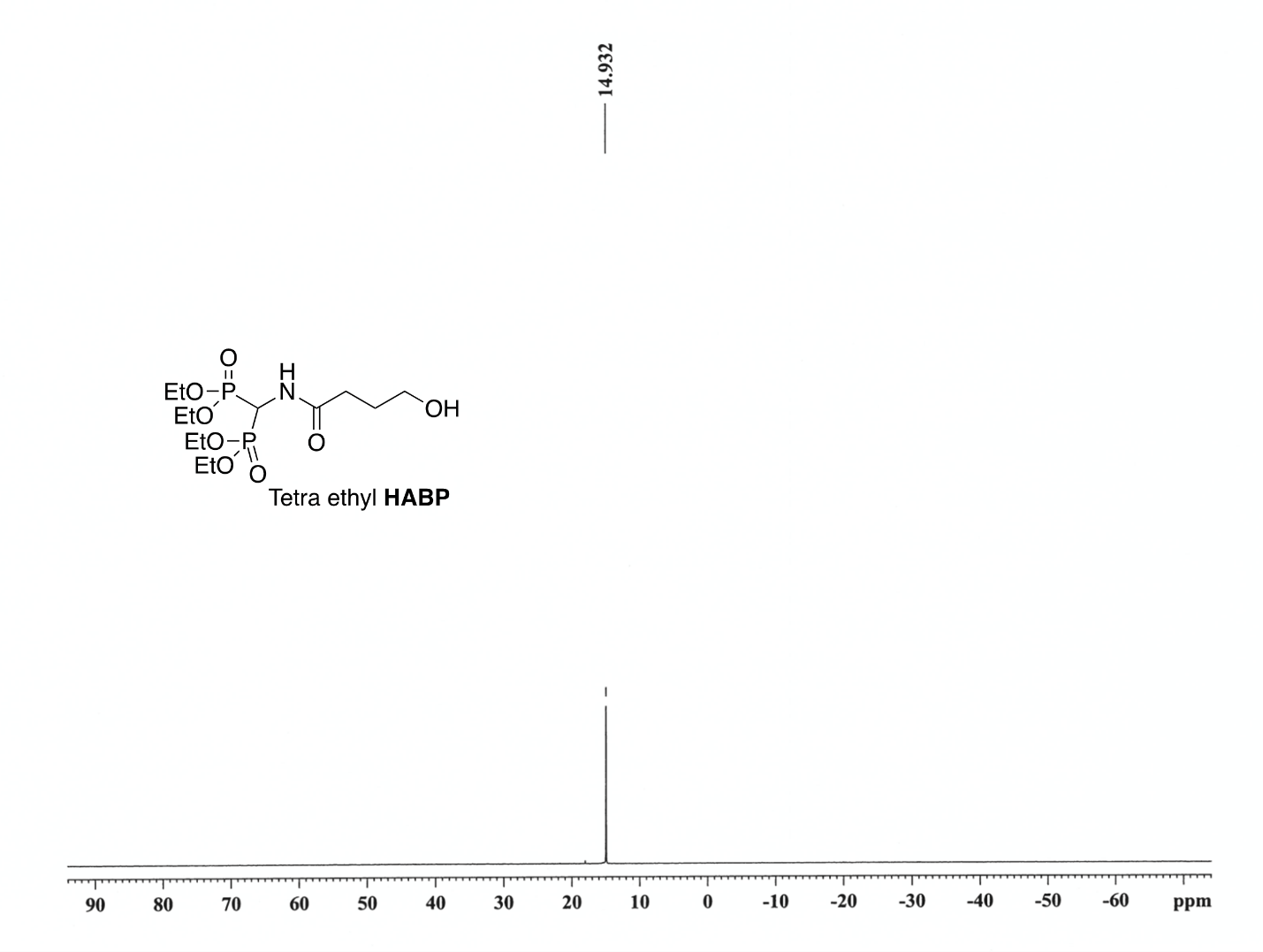


^31^P NMR Spectrum (CD_3_OD)


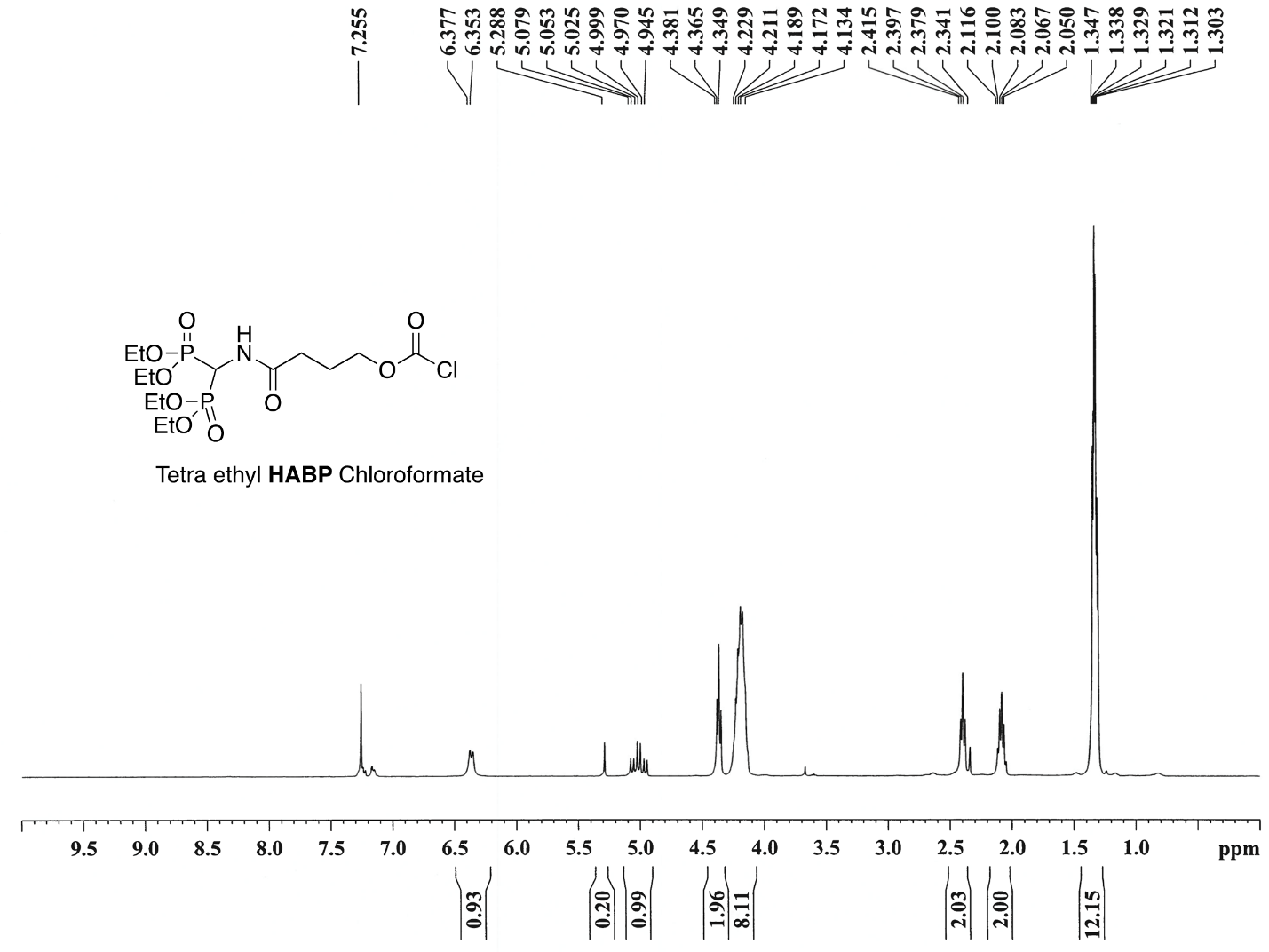


^1^H NMR Spectrum (CDCl_3_)


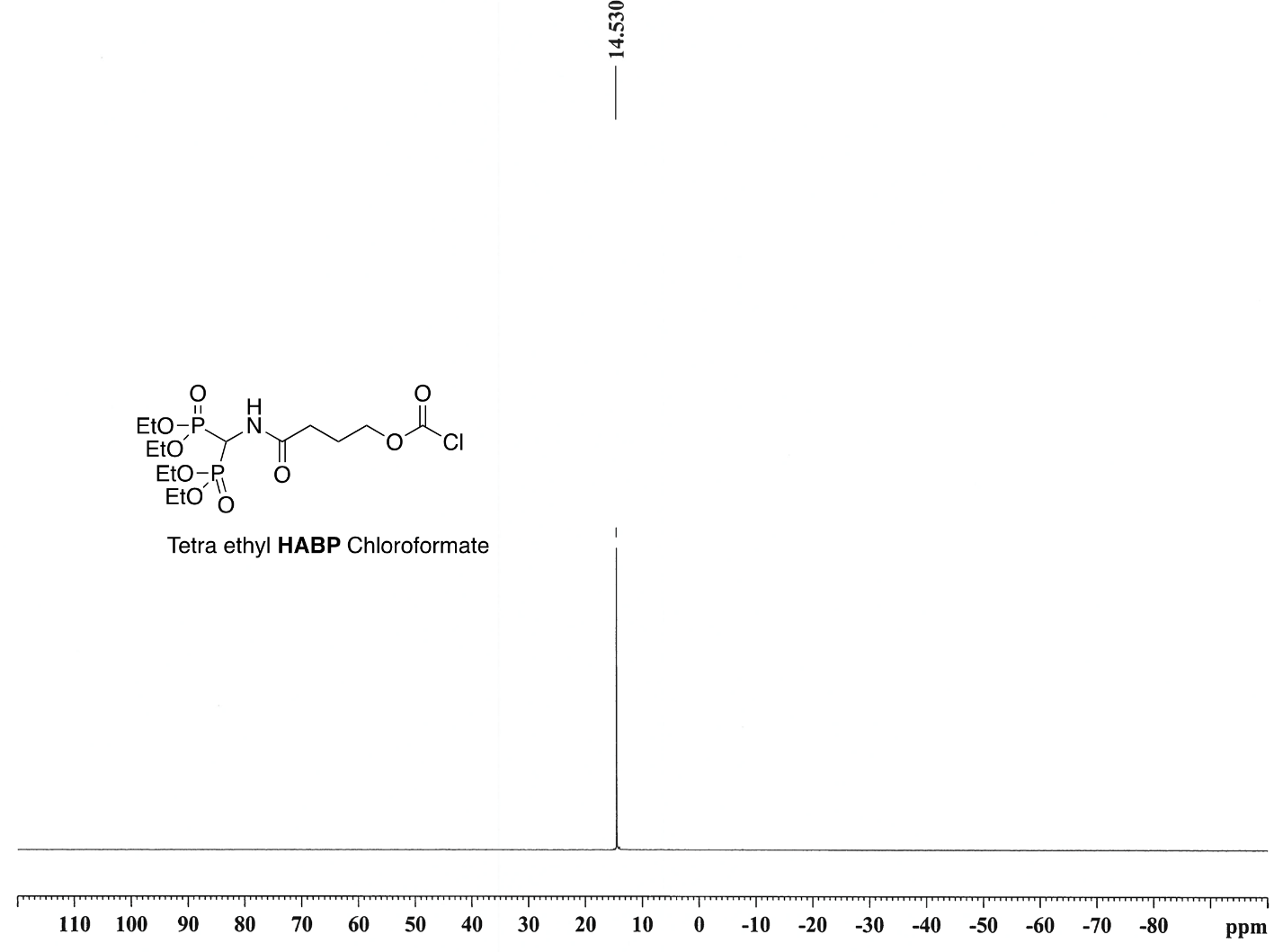


^31^P NMR Spectrum (CDCl_3_)

^1^H NMR Spectrum (D_2_O)

^13^C NMR Spectrum (D_2_O)


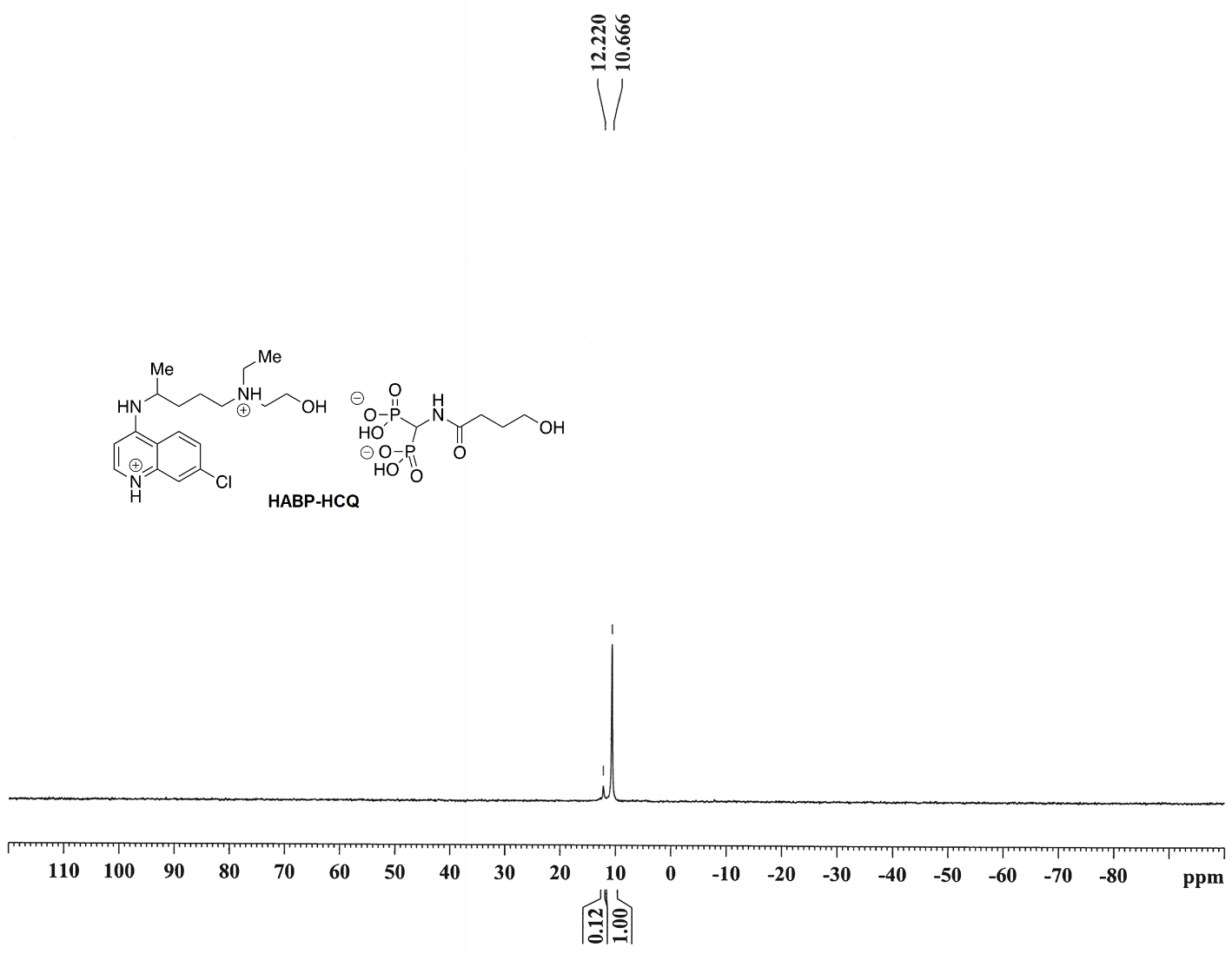


^31^P NMR Spectrum (D_2_O)
